# Procalcitonin in the Post-Operative Burn Patient

**DOI:** 10.3390/ebj4040040

**Published:** 2023-11-16

**Authors:** Ludo Masole, Chikwendu J. Ede, Adelin Muganza

**Affiliations:** Department of Surgery, Chris Hani Baragwaneth Academic Hospital, University of the Witwatersrand, Johannesburg 1864, South Africa

**Keywords:** procalcitonin, burn, debridement, surgery, sepsis, infection, South Africa

## Abstract

Serum procalcitonin (PCT) is a biomarker used to diagnose sepsis and infection. Following invasive bacterial infection, PCT is detectable in peripheral blood. The aim of our study was to determine if there is a relationship between serum PCT post-burn wound debridement and burn-related sepsis. In total, 34 participants were recruited from 1 November 2019 to 31 July 2020. Serum PCT levels were drawn on days 0, 1, 2, and 3, with day 0 being the day of the surgery. Blood culture samples were drawn on days 0 and 3. Statistical analyses were performed using STATA©. Descriptive statistics were presented as the median for continuous data and frequencies for categorical data. A two-sample Wilcoxon–Mann–Whitney test was performed to assess the correlation between the PCT values and blood culture positivity. In all, 33 burn debridement procedures were completed, and 1 patient demised before surgery. The median age was 35.5 years; 61.8% were male. Four patients had comorbidities. There was a trend of higher PCT values from day 0 to day 3. The median PCT on day 0 was 3.30 µg/L (IQR 0.78–15.10), compared to day 3 PCT which was 5.15 µg/L (IQR 1.35–18.55). The median values for serum PCT for days 0 to 3 were above the normal threshold regardless of BC positivity. There was a statistically significant difference in the PCT levels between positive and negative BC, with a *p* value of 0.0087 for day 3 serum PCT. The findings indicate an association of a high serum PCT level with a positive blood culture in a burn patient post-debridement surgery. A higher numerical threshold/cut-off of serum PCT should be used for this cohort of patients, to aid in the diagnosis of sepsis. A cut-off value could not be determined due to the small sample size.

## 1. Introduction

Burns are a major public health challenge in Sub-Saharan Africa, and a cause of significant morbidity and mortality. The Global Burden of Disease 2019 study reported more than 8 million new cases of burns globally, with a disproportionate impact on low-to-middle income countries [1]. Burn prevention remains the cornerstone of reducing the burden of burn injury on society. Prevention strategies in low-to-middle income countries (LMIC) often are lacking, with little to no epidemiological data to direct a coordinated public health strategy to address this problem [2,3]. A unique challenge in the management of hospitalized burn patients is the additional insult of wound debridement in a patient with a hyper inflammatory state [4,5]. The local and systemic effects resulting from the burn injury weaken the entire immune system with consequent susceptibility to infection [6,7]. The incidence of sepsis in hospitalized burn patients is estimated to be 8–42.5% and a documented mortality of 28–85% within this subgroup of patients [4,8,9]. Burn sepsis is the main contributor to burn-related mortality. Procalcitonin (PCT); a 116-amino acid pro-hormone produced by the parafollicular c-cells of the thyroid gland and certain neuro-endocrine glands has emerged as a biomarker for sepsis. Under normal physiological conditions, PCT is not detectable in peripheral blood; however, following invasive bacterial infection, it is detectable [10,11]. Peripheral blood levels of PCT in excess of 0.5 ng/mL are regarded as abnormal [10]. PCT seems to outperform more traditional biomarkers in the severely burned patient in identifying sepsis [12]. The degree of PCT elevation in the context of trauma is possibly dependent on the severity of the surgical trauma, suggesting a need for a higher threshold to be used in this group of patients [13,14,15,16]. In their prospective study, Mathur and colleagues demonstrated that while major trauma does cause serum PCT elevation, the PCT trend can be used as a surrogate marker in the early diagnosis of sepsis [17]. Serial PCT measurements were further demonstrated to be useful in identifying low-risk patients post-major elective abdominal surgery [18]. Liu et al. investigated the effectiveness of PCT as a biomarker in extensive burns, and concluded that PCT is useful in prognostication and is an early harbinger of burn-related sepsis [19]. There are few data regarding the trend of serum PCT in the peri-operative burn patient, with only one retrospective review identified in the literature [20]. The aim of our study was to determine if there is a relationship between serum PCT post-burn wound debridement and burn-related sepsis. The primary objective was to determine serum PCT level threshold post-burn wound debridement that correlates with a positive blood culture, and secondarily to determine factors that predict elevated serum PCT post-burn wound debridement.

## 2. Materials and Methods

### 2.1. Study Setting

The study was undertaken at Chris Hani Baragwaneth Academic Hospital (CHBAH), in the Adult Burns Unit (ABU), Johannesburg, South Africa, which is a middle-income country. This is a 22-bed Level-1 burn unit, which receives referrals from Gauteng as well as three other neighbouring provinces. For all patients, on admission, a panel of blood tests is taken, which includes a baseline serum procalcitonin (PCT). Those ABU patients requiring critical care have routine daily PCT levels taken, while daily testing is not conducted in those not requiring critical care. In addition, a blood culture (BC) and PCT level are part of the panel of investigations taken if infectious complications or sepsis are suspected.

The ABU has a hospital infection prevention and control policy. The policy was informed by guidelines recommended by the World Health Organization (WHO) and the International Society of Burn Injury (ISBI) [21,22]. In the ABU intensive care section, each patient is nursed by a dedicated nurse, in a single isolation room. Before each admission, a fogging machine is used to clean and disinfect the area. It is a requirement that the nurse wears a plastic apron, gown, cap, gloves, and face mask when nursing a patient. There is an information chart at the entrance of the unit demonstrating hand hygiene, which is compulsory for each visitor to follow. An infection control steward is nominated on a weekly basis to ensure staff and visitor compliance to the unit infection control standards. Additionally, frequent in-house training is part of the unit policy to reinforce infection control principles.

### 2.2. Inclusion and Exclusion Criteria

The populations included in this study were patients aged 18 years and above with a total body percentage area (TBSA) burn of 15% or more and with an expected length of stay of more than 5 days. Consecutive patients admitted from 1 November 2019 to 31 July 2020, who were planned for surgical debridement, were included in the study. Informed consent was obtained from the patient or their next of kin where the patient was unable to provide informed consent. Exclusion criteria were refusal or withdrawal of consent.

### 2.3. Study Design

This was a prospective observational study. Purposive sampling was used for consecutive patients admitted during the eight-month study duration. In total, 34 participants were enrolled into the study. A positive blood culture was used as a surrogate for burn sepsis in this study. The day of surgery was designated as day 0, then the first post-operative day as day 1, and so forth until day 3. A serum procalcitonin, reported in micrograms/litre (µg/L), was measured pre-operatively on the day of the surgery, and on days 1, 2, and 3. A blood culture was performed on the designated day 0 before surgery and day 3 in a sterile fashion. The procedure for the collection of a sample for blood culture was to don a face mask and cap, and then an aseptic handwash was completed using chlorhexidine soap. Sterile gloves and gowns were worn. The site for collection was cleaned with betadine, allowed to dry, and 10 mL of blood collected, which was equally divided between 1 aerobic bottle and 1 anaerobic bottle as per the ABU unit policy. Only the first surgical debridement was considered. All samples were analysed at the National Health Laboratory Services (NHLS). A quantitative serum PCT was measured using a Cobas^®^ 8100 (Roche Diagnostics (Hoffman), Basel, Switzerland). The following thresholds in µg/L applied for serum PCT for this assay: <0.5 systemic infection unlikely/localized infection, 0.5–2 systemic infection possible, 2–10 suggestive of systemic infection, >10 severe systemic infection/septic shock. The blood culture sample was run and analysed using the BacT/ALERT 3D™ Microbial Detection System (bioMérieux, Duram, NC, USA).

### 2.4. Data Collection

Informed consent was obtained and signed by the patient or their next of kin. A data collection sheet was created and filled in. Data collected were entered into a Microsoft^®^ Excel^®^ 2016 (Microsoft Corporation, Redmond, WA, USA) spreadsheet. Each patient data collection sheet was de-identified and assigned a unique code. The demographic information collected included age, gender, and existing medical comorbidities. Variables of data collected were burn depth, type, total body surface area (TBSA) burn, presence of inhalation injury, type of surgery performed, TBSA operated, day post-burn, time on operating table, as well as the use of prophylactic and/or treatment antibiotics. Blood culture results were recorded as either positive or negative for day 1 and D3, respectively. The serum PCT value was recorded for each day as µg/L.

### 2.5. Statistical Analysis

Statistical analyses were performed using STATA© (version 16.1 suite of analysis software STATA© LLC; College Station, TX, USA). Descriptive statistics were presented as median (interquartile range—IQR) for continuous data and frequencies for categorical data. The median value and interquartile range of the PCT level were reported for each day in µg/L. A two-sample Wilcoxon–Mann–Whitney test was performed to compare the PCT values on day 0 and day 3 with positivity of any blood culture. A test for statistical significance was considered at a threshold *p* value of less than or equal to 0.05.

### 2.6. Ethics

Ethics clearance for this prospective observational study was obtained from the Human Research Ethics Committee (HREC) of the University of Witwatersrand; Certificate No: M181119.

## 3. Results

In total, 34 participants were recruited into the study from 1 November 2019 to 31 July 2020. Five (5) participants approached to participate did not provide consent and were excluded from the study. One participant demised on the day of the surgery. Of the 33 participants, three did not have pre-operative blood culture results as the specimens were rejected at the laboratory. Additionally, six (6) day 3 blood culture results were not available due to clinician error in the collection and/or labelling of specimens. The baseline characteristics of the study participants are as illustrated in Table 1. Only four patients had comorbidities. The types of comorbidities identified in our study were HIV infection, hypertension, type 2 diabetes mellitus, and epilepsy. The median age of participants was 35.5 years, with the majority being male. Inhalation injury was diagnosed on clinical suspicion. A total of 33 procedures were completed. These were 26 surgical debridements conducted without temporary skin substitute cover. Seven surgical debridements were conducted with temporary skin substitute cover: five of these with porcine xenograft and two with a combination of porcine xenograft plus Nanotrix™ (Stellenbosch Nanofiber Company, Cape Town, South Africa).

The earliest day a post-burn injury wound debridement was conducted was on day 2 post-burn injury, with the latest being day 18. The median day post-burn injury for surgery was day 5 post-burn injury. The burn wound total body surface area involved in the burn injury and operative parameters are as shown in Table 2.

Gram-positive and Gram-negative organisms were cultured for both the pre-operative (day 0) and the post-operative (day 3) blood cultures as illustrated in Table 3. For day 0 BC, the most cultured organism was *Acinetobacter baumanii*, whilst that for day 3 BC was *Staphylococcus aureus*. On day 0, the frequency of a polymicrobial growth on blood cultures was four samples, and that on day 3 was similarly four samples. For each blood culture with polymicrobial growth, day 0 samples all had two or more microorganisms identified. Day 3 samples with polymicrobial growth had only samples culturing more than two microorganisms.

**Figure 1:**
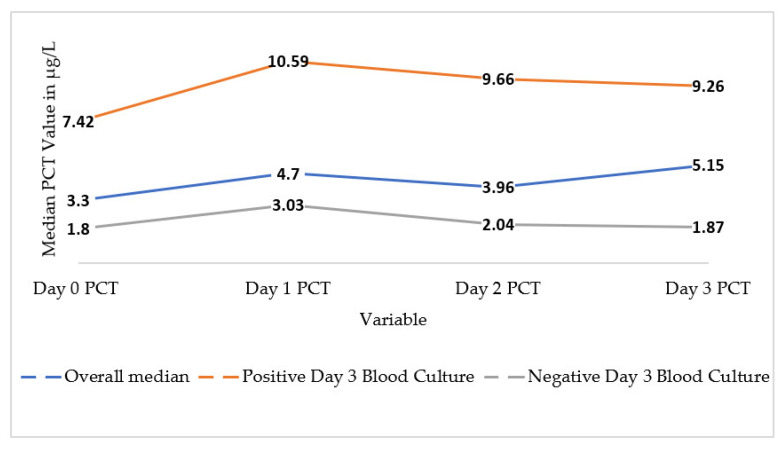
Overall median PCT values from day 0 to day 3 in µg/L and median PCT values compared to blood culture result on day 3.

There was a trend towards higher serum PCT values from day 0 to day 3. The median PCT on day 0 was 3.30 (IQR 0.78–15.10), compared to day 3 PCT which was 5.15 (IQR 1.35–18.55) as demonstrated in Figure 1. The median PCTs for days 1 and 2 were also elevated above the threshold, with an IQR of 0.78–17.11 and 0.92–9.95, respectively. All median values for serum PCT for days 0 to 3 were above the threshold considered to be within normal limits. These median values were also above 2.0 µg/L, the threshold level indicating the possibility of systemic infection. Also demonstrated in Figure 1 is the median PCT level for patients according to their blood culture result on day 3. The median values for each day were higher for those patients who had a positive blood culture compared to those with a negative one.

A two-sample Wilcoxon–Mann–Whitney test was performed to compare the serum PCT level at day 0 and day 3 with the blood culture result (negative and positive). In the analysis for day 3, the blood culture result considered was for either day 0 or day 3. If a blood culture on either day had been positive, this was included in this analysis. As shown in Table 4, this demonstrated a statistically significant difference in the PCT level between positive and negative BC. The median serum PCT on day 0 and day 3 in the subgroup of patients with a negative BC was 1.8 and 0.91, respectively. Both values were above the normal serum PCT threshold of <0.5 µg/L. The serum PCT on both days 0 and 3 was above the normal serum PCT threshold regardless of BC positivity.

A two-sample Wilcoxon–Mann–Whitney test was conducted to compare the serum PCT level at day 2 compared with the day 3 blood culture results. Table 5 shows that while the median PCT level was higher in those with a positive blood culture compared to those with a negative blood culture, this value lacked statistical significance (*p*-value 0.0710).

A post-hoc analysis utilizing a two-sample Wilcoxon–Mann–Whitney test was conducted for day 0 and day 3 PCT compared to blood culture results with microorganisms known to be skin contaminants re-categorized as negative blood culture. These microorganisms included *staphylococcus epidermidis* and *coagulase-negative staphylococcus.* For day 0 PCT, the median PCT was 1.12 (IQR 0.42–4.23) for those with a negative blood culture, and 21.35 (IQR 7.42–62.62) for those with a positive blood culture. The calculated *p*-value was 0.002, indicating a stronger statistical significance compared to when these organisms were categorized as a ‘positive blood culture’. Similarly, the results for day 3 PCT demonstrated a median PCT of 1.83 (IQR 0.61–3.78) for the negative blood culture and 14.34 (5.19–32.58) for the positive blood culture. Statistical significance was reached with a calculated *p* value of 0.0038.

## 4. Discussion

This prospective study to investigate the utility of serum PCT in the post-operative burn patient showed that the median serum PCT in burn patients is higher than the normal threshold. This is consistent with what has been reported in the literature [19,23]. In our study, the median PCT was above the threshold both pre-operatively and post-operatively. This included even the subgroup of patients with negative BC, suggesting the trauma of the burn injury itself causes an induction of PCT. If the current normal threshold for serum PCT is applied to diagnose burn sepsis, this may result in unnecessary exposure to antibiotics, thereby encouraging antibiotic resistance. There has been a suggestion that a different threshold PCT for normal be used in burns and trauma patients [13,14,15,16,19], with no consensus as to what exactly this figure should be. In the pre-operative burn patient with a negative blood culture, the median serum PCT level in our study was 1.8µg/L. When the BC was positive, the median PCT level was 8.47 and 8.35 on day 0 and day 3, respectively. This indicates that a higher threshold could be used in burns and trauma patients. Due to the small sample size in our study, we cannot make a recommendation from our results as to what this higher threshold to be used in burns and trauma patients should be.

In our study, sepsis was defined by blood culture positivity. A positive blood culture was defined by an identified organism, regardless of the identity of the microorganism. This contrasts with clinical practice. In most units, including ours, the American Burns Association 2007 consensus definition for burn-related sepsis is used. The ABA 2007 consensus takes into consideration many other clinical parameters [24]. In addition, some organisms such as *Coagulase-negative staphylococci* are considered contaminants from the skin, unless other clinical parameters as defined in the ABA 2007 are also present. It is difficult to accurately diagnose burn-related sepsis since criteria included by the ABA and other society guidelines are like the systemic inflammatory response syndrome (SIRS) related to the burn injury itself. The Surviving Sepsis After Burns Campaign 2023 consensus explicitly recommends against the use of SIRS criteria to diagnose sepsis in burn patients [25]. A parameter such as serum PCT can be added to the current definitions of sepsis to augment the accuracy of making the diagnosis.

A statistically significant association of high PCT and BC positivity on either day 0 or day 3 was observed in our study. This was more significant when correlated specifically with day 3 BC positivity and underscores the utility of serum PCT in identifying or suggesting sepsis in the burn patient. Day 2 PCT also had a statistically significant association with a positive blood culture on day 3. This has been suggested in several retrospective [9,19,20] and prospective [12] studies as well as a meta-analysis [26]. Conversely, some data suggest that serum PCT is not a precise indicator of sepsis at the time of diagnosis [27]. However, these studies did not take into consideration the impact that surgery has on PCT utility. Our study suggests that serum PCT can still be used in the patient who has sustained a double hit of both burn trauma and surgery. While it was a retrospective study, Cabral and colleagues in their 2018 study demonstrated that PCT can reliably identify sepsis in the burn patient undergoing surgery [20]. They considered several surgeries, primarily escharotomies, skin grafts and flaps, as well as digit/limb amputations, and we considered only surgical debridement. An assumption can be made that our findings can be applicable to other surgeries on burn patients as well. The Cabral study was retrospective and conducted in a high-income country setting, where the rate of burn-related sepsis is far less than that found in low- and middle-income countries. The findings of our study are more applicable to the LMIC setting, as no prospective study examining this has, to the best of our knowledge, been conducted prior to ours.

The stronger statistical significance of high serum PCT in our study was more evident in the post-operative burn patient who had a post-operative positive blood culture. This is somewhat congruent with the findings of Cabral and colleagues [20], as they identified that the highest serum PCT values in their study were those who had pre-operative as well as post-operative sepsis. In our study, this statistically significant association was evident even after re-classifying the blood cultures that grew skin contaminants as negative rather than positive. This suggests that PCT is induced with pathogenic organisms in the blood stream. In addition, a single PCT value has limited value; therefore, serial measurements need to be conducted.

Our study suggests that a higher threshold for serum PCT in this group of patients may be required. Several studies have attempted to determine this value with ranges of more than 1 ng/mL to 8.5 ng/mL [19,20,23], indicating the lack of consensus on this issue. The usefulness of a threshold is to identify low-risk patients for possible early discharge [19], as well as a surrogate marker for prognostication of the development of infectious complications as well as mortality [9,20,23]. In our study, the median value for patients with negative blood cultures pre-operatively on day 0 and day 3 was 1.8 µg/L and 0.9 µg/L, respectively. A recommendation for a threshold could not be made in our study due to the small sample size. A larger study could be done to definitively establish a serum PCT threshold that can be used. This threshold value can then be used in conjunction with other burn sepsis criteria to assist in diagnoses of burn-related sepsis.

Several factors have been suggested to correlate with elevated PCT levels. These include full thickness TBSA burns, burn index, severe inhalation injury, as well as the Sequential Organ Failure Assessment (SOFA) score [19]. In our study, we did not investigate these factors and therefore cannot comment about such an association. The heterogeneity of data limited the ability to perform a trend analysis of the kinetics of PCT post-debridement surgery.

To the best of our knowledge, this is the first study of its kind conducted in a LMIC setting. The findings pave the way for a larger, more robust study to confirm the utility of PCT in the burn patient who undergoes surgery. A larger, prospective study may also determine a threshold that should be used in this specific group of patients.

This study has several limitations. The study was performed in a specialized burn unit with strict admission criteria and was observational. This may have introduced selection bias. These findings may have limited applicability for burn patients that were not managed in a specialized burn unit, which was the case for several patients managed in our referral drainage area. Units that manage burn injuries used criteria outlined in the American Burns Association 2007 consensus definition of sepsis, or other clinical parameters rather than only BC positivity. Defining sepsis as a positive blood culture was a limitation of our study as it restricted the applicability of our findings in the non-research setting. In addition, for the purposes of this study, a surveillance BC at day 0 was taken. This study may have classified patients with bacteraemia as having sepsis, even though they did not meet the criteria for sepsis according to the 2007 ABA consensus definition. This in turn may inadvertently lead to the overexposure of antibiotics. The sample size was small, limiting the ability of the study to establish a threshold value for serum PCT as well as identifying factors which correlated to elevated PCT levels in the study population. A study with a larger sample size is required. There are limited studies conducted in LMIC investigating the use of serum PCT in this population. This may be due to financial constraints. More studies, with larger sample sizes and more findings conducted in a prospective manner are required in this area.

## 5. Conclusions

This prospective study demonstrates that burn patients have a higher-than-normal threshold serum PCT. It also demonstrates the association of a high serum PCT level with a positive blood culture in a burn patient post-debridement surgery. The usefulness of serum PCT in these patients has been demonstrated and the fact that PCT is a serum biomarker that can be used in this specific group. Burn surgery itself causes an induction in PCT; therefore, in these patients, the interpretation of elevated serum PCT must be conducted with caution. Caution will prevent unwarranted antibiotic exposure, a contributor of antibiotic resistance. The serum PCT level in burn patients with or without a positive blood culture is higher than the threshold considered positive in other patients. A higher threshold should be used for this cohort of patients, to aid in the diagnosis of infectious complications to augment already existing criteria used to diagnose burn-related sepsis. A cut-off value could not be determined due to the small sample size, and further studies need to be designed with enough statistical power to establish this value.

## Figures and Tables

**Table 1:** Characteristics of participants.

	Number	Percentage (%)
Gender		
Males	21	61.8
Females	13	38.2
Comorbidities		
Yes	4	11.8
No	30	88.2
Burn depth		
Partial	28	82.4
Deep	6	17.6
Burn type		
Electrical	4	12.0
Flame	25	73.3
Hot liquid/solid	5	14.7
Inhalation injury		
Yes	10	29.5
No	24	70.5

**Table 2:** Operative burn parameters.

Parameter	Median	Interquartile Range
**TBSA %**	34%	20–40%
**TBSA % operated**	20%	20–30%
**Time on operating table**	105 min	90–155 min

**Table 3:** Organisms identified on blood cultures.

Day 0 Blood Culture	Frequency	Day 3 Blood Culture	Frequency
*Acinetobacter baumanii*	5	*Staphylococcus aureus*	5
*Coagulase-negative staphylococci*	4	*Klebsiella pneumoniae*	4
*Proteus mirabilis*	3	*Acinetobacter baumanii*	3
*Pseudomonas aeruginosa*	3	*Coagulase-negative staphylococci*	2
*Klebsiella pneumoniae*	3	*Proteus mirabilis*	1
*Enterobacter cloacae*	2	*Pseudomonas aeruginosa*	1
*Enterococcus faecalis*	2	*Enterococcus faecalis*	1
*Stenotrophomonas maltophilia*	1	*Achromobacter xylosoxidas*	1
*Streptococcus agalactiae*	1		
*Staphylococcus epidermidis*	1		
*Escherichia coli*	1		

**Table 4:** Serum PCT level compared to day 0 and day 3 blood culture results: ^ = µg/L.

Variable	Positive Blood Culture	Negative Blood Culture	*p* Value
**PCT Day 0** **Median (IQR) ^**	*n* = 15 8.47 (0.78–31.88)	*n* = 15 1.8 (0.64–4.23)	0.0161
**PCT Day 3** **Median (IQR) ^**	*n* = 18 8.35 (2.83–23.14)	*n* = 9 0.91 (0.61–2.68)	0.0087

**Table 5:** Serum PCT level compared to day 0 and day 2 blood culture results: ^ = µg/L.

Variable	Positive Blood Culture	Negative Blood Culture	*p* Value
**PCT Day 2** **Median (IQR) ^**	*n* = 11 9.66 (3.37–12.31)	*n* = 11 2.04 (0.37–5.69)	0.0710

## Data Availability

The data presented in this study are available on request from the corresponding author. The data are not publicly available due to privacy concerns.
